# Invasive squamous cell carcinoma on sternotomy scar

**DOI:** 10.1002/ccr3.8655

**Published:** 2024-03-27

**Authors:** Nima Sarisarraf, Farnaz Araghi, Zahra Asadikani, Hamideh Moravvej Farshi

**Affiliations:** ^1^ Rajaie Cardiovascular Medical and Research Center Iran University of Medical Science Tehran Iran; ^2^ Skin Research Center Shahid Beheshti University of Medical Sciences Tehran Iran

**Keywords:** Marjolin ulcers, scars, squamous cell carcinoma, sternotomy

## Abstract

Early detection and management of skin tumors has significant importance due to their potency to metastasize. Hence, this study recommends raising the patients' awareness about chronic ulcers and the potential alterations they may experience.

## 1 INTRODUCTION

Since 1828, the term Marjolin's ulcer has been referred to as squamous cell carcinoma (SCC) which grows on the burned ulcers. Currently, the terminology has been revised to include all types of skin tumors that develop on the damaged skin not just burn scars.^1^ Moreover, it is believed that the most common skin cancer that develops on these types of scars is SCC.^2^

In addition, this damaged skin would consist of infected wounds, sinuses, and long‐lasting scars.^3^ However, there are only a few studies in which the SCC grew on surgical uncomplicated scars particularly sternotomy surgical scars.

Here, we are reporting a patient in which an invasive SCC tumor developed on the uncomplicated sternotomy scar after 5 years.

## 2 CASE HISTORY

An 85‐year‐old male patient was referred to our dermatologic clinic with an ulcerated lesion on his chest. He mentioned the rapid growth of the tumor within 2 months on his surgical scar of the chest. He didn't complain of pain or bleeding.

He had a history of pelvic bone fracture related to the trauma 5 months prior. Additionally, he had undergone an uncomplicated coronary artery bypass grafting (CABG) 5 years earlier. His sternotomy scar on his chest had been excellently healed 1 month later and he denied any trauma or infection in this area.

His medical history was negative for any other comorbidities.

**FIGURE 1:**
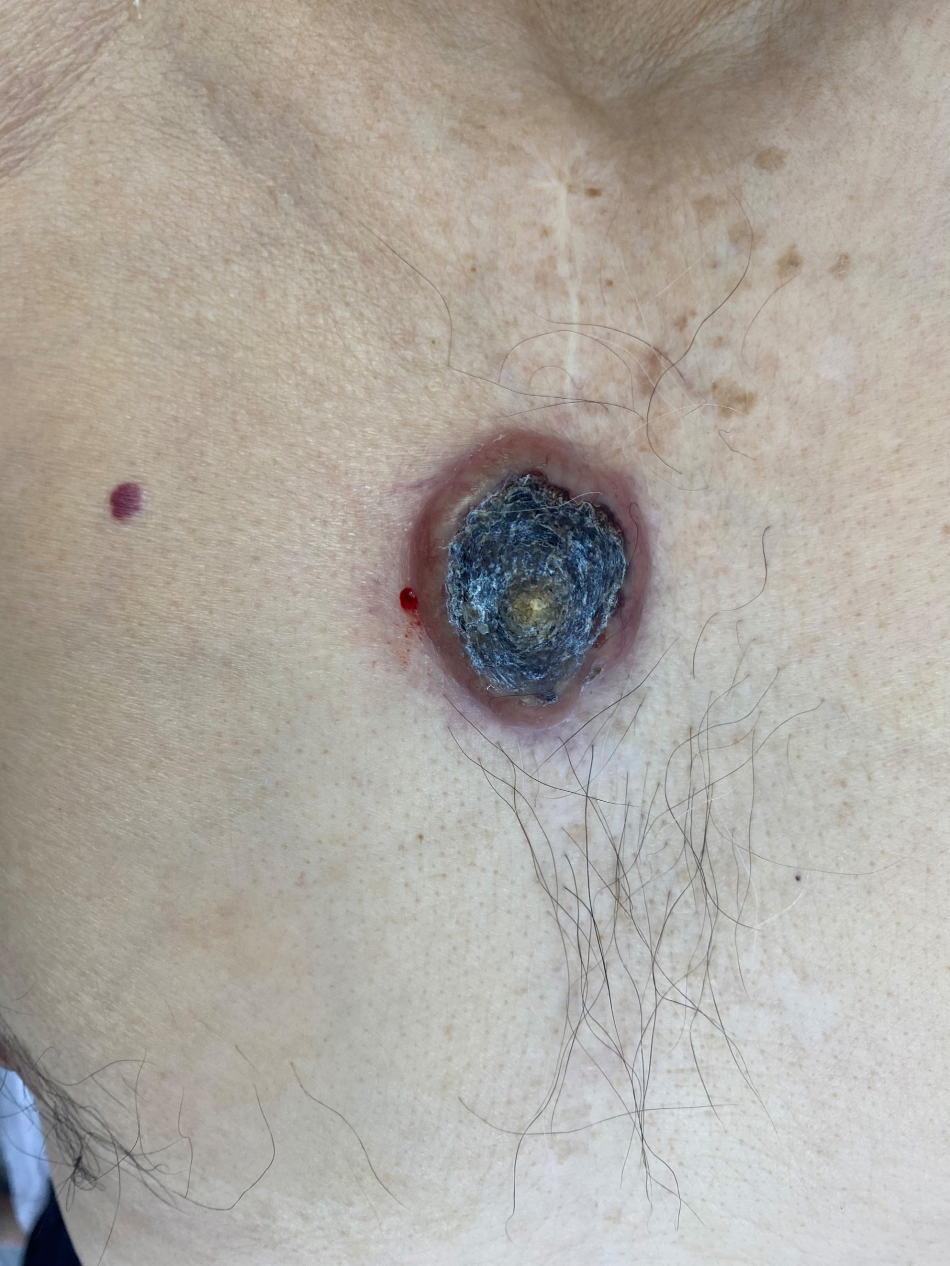
An ulcerated nodule on the middle line of the chest, exactly in the middle of his sternotomy scar, with 3‐cm diameter.

In his physical examination, an ulcerated nodule was observed on the middle line of the chest, exactly in the middle of his sternotomy scar. The nodule had a 3‐cm diameter and the surrounding skin of the nodule was indurated (Figure 1). The center of the nodule was a crater full of keratin. No palpable lymph node has been detected during the examination.

## 3 METHODS

He had a chest CT scan that showed cardiomegaly and a ground glass appearance in the lower part of the lungs which were suggestive of chronic heart failure.

Skin biopsy was done, considering differential diagnoses such as keratoacanthoma and squamous cell carcinoma.

**FIGURE 2:**
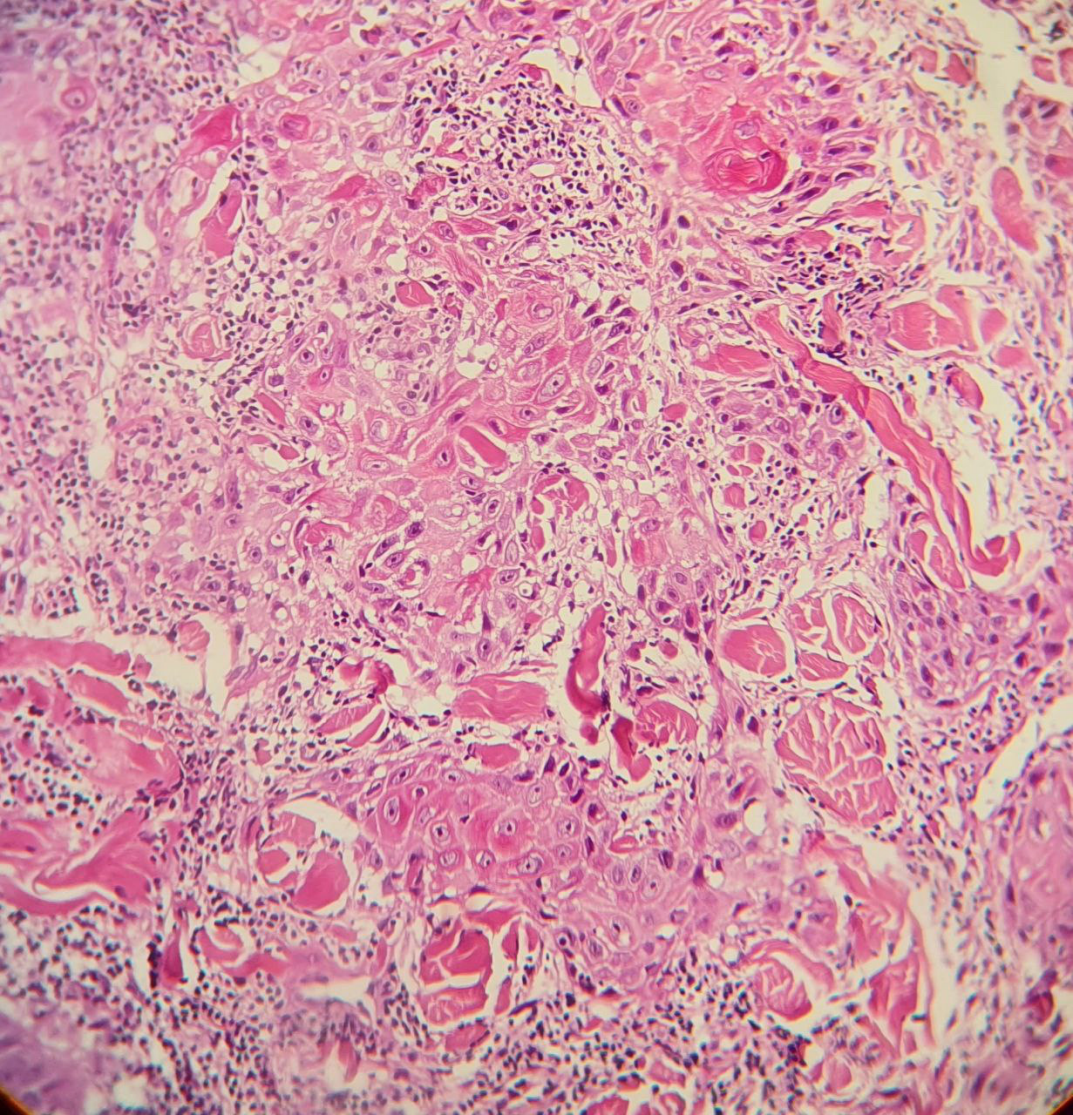
Endophytic crateriform squamous hyperplasia with severe dysplasia and evidence of dermal invasive consistent with invasive squamous cell carcinoma, crateriform (×40).

In his microscopic examination (Figure 2), endophytic crateriform squamous hyperplasia with severe dysplasia and evidence of dermal invasive consistent with invasive squamous cell carcinoma, crateriform.

## 4 RESULTS

Due to the general condition of the patient, we decided to refer him to the plastic surgery hospital to perform the surgery using general anesthesia and precise monitoring.

Subsequently, the specimen was sent to the pathobiology laboratory for a detailed analysis of the margins, which confirmed the absence of any tumor.

## 5 DISCUSSION

This study presents a patient with an invasive SCC tumor on his surgical scar which had previously healed 5 years ago. Previous research identified that the lower extremity, particularly the volar side of the foot, is the most common area for such tumors. Primarily, in 1991, Korula et al. reported a similar case of an SCC tumor that grew on the sternotomy scar after 12 months.^4^ Furthermore, there have been nearly five cases in which SCC has been detected on the scar resulting from abdominal surgery.^5^ Consequently, the trunk is the least frequent location for such occurrences' identical to our case report.^1^

The potential reason for this trend could be the intensity of sun exposure to the extremities rather than the trunk. Additionally, it is assumed that sun exposure could play a significant role in squamous carcinoma growth in individuals with fair skin like our patient; however, other factors that result in chronic ulcers like infectious, discoid lupus erythematosus, and HIV infection could be responsible in the individuals with darker skin.^6^

Generally, these Marjolin ulcers have been categorized into acute and chronic types depending on the time period between the first injury and the malignancy appearance on the injured skin. A recent investigation revealed that the average duration for this process is approximately 5 years. Therefore, the majority of these Marjolin ulcers are categorized as chronic type.^1^

The pathogenesis of these chronic types could be related to the ongoing insults and irritations to the scar tissue associated with the lack of vascularization, resulting in decreasing the local immunity of the area and the tumor growth.^6^

In this report the tumor lesion was displayed within 2 months; however the surgical scar had healed 5 years prior. Based on recent studies, the possible explanation for such rapid occurrence could be attributed to the genetic factors that make certain individuals more susceptible to tumor growth.^1^ Accordingly, p53 over‐expression, E‐cadherin and beta‐catenin decline may lead to the aggressive behavior of SCC in an individual.^7^ As indicated previously, SCC tumors that develop on scars have the potential to metastasize in 10–100 of the patients, whereas this may occur in only 1% of the patients with nonscar‐SCC tumors.^8^

Taken together, early detection and management of such tumors hold remarkable significant importance among patients. Hence, this study recommends raising the patients' awareness about chronic ulcers and the potential alterations they may experience. Furthermore, it emphasizes the need for accurate examination of surgical ulcers with any changes even after the healing process completion.

## AUTHOR CONTRIBUTIONS

**Nima Sarisarraf:** Conceptualization; writing – original draft; writing – review and editing. **Farnaz Araghi:** Conceptualization; data curation; investigation; supervision; validation; writing – original draft; writing – review and editing. **Zahra Asadikani:** Investigation; project administration. **Hamideh Moravvej Farshi:** Supervision.

## FUNDING INFORMATION

None.

## CONFLICT OF INTEREST STATEMENT

The authors have no conflict of interest to declare.

## ETHICS STATEMENT

The ethical issues were completely considered to prepare this case report according to our institution's ethical board guidelines. Moreover, this article was prepared regarding the declaration of Helsinki.

## CONSENT

Written informed consent was obtained from the patient to publish this report in accordance with the journal's patient consent policy.

## Data Availability

Data openly available in a public repository that issues datasets with DOIs.
